# Efficacy of Intravenous Hydrocortisone Treatment in Refractory Neonatal Seizures: A Report on Three Cases

**DOI:** 10.3390/brainsci10110885

**Published:** 2020-11-20

**Authors:** Gabriella Di Rosa, Daniela Dicanio, Antonio Gennaro Nicotera, Patrizia Mondello, Laura Cannavò, Eloisa Gitto

**Affiliations:** 1 Unit of Child Neurology and Psychiatry, Department of Human Pathology of the Adult and Developmental Age, “Gaetano Barresi” University of Messina, 98125 Messina, Italy; daniela.dicanio.7@gmail.com (D.D.); antonionicotera@hotmail.com (A.G.N.); 2 Unit of Hematology, Department of Human Pathology of the Adult and Developmental Age, “Gaetano Barresi” University of Messina, 98125 Messina, Italy; patrizia.mondello@unime.it; 3 Neonatal Intensive Care Unit, Department of Human Pathology of the Adult and Developmental Age, “Gaetano Barresi” University of Messina, 98125 Messina, Italy; laura_cannavo@hotmail.it (L.C.); egitto@unime.it (E.G.)

**Keywords:** hydrocortisone, neonatal seizure, neuronal inflammation, neonatal status epilepticus

## Abstract

Neonatal seizures are the most common neurological emergency, and neonatal status epilepticus (NSE) remains a controversial entity, with no general consensus about its definition and treatment. Here, we report on three newborns with NSE refractory to first- and second-line antiepileptic drugs successfully treated with intravenous (IV) hydrocortisone. The patients had previously failed therapy with levetiracetam, phenobarbital and midazolam, showing persistent clinical and electrical seizures. Modulation of brain inflammation triggered during prolonged epileptic activity has been thought to potentially explain the beneficial effects of anti-inflammatory treatment.

## 1. Introduction

Despite neonatal seizures representing the most common neurological emergency, occurring in 1 to 3 per 1000 live births [1], neonatal status epilepticus (NSE) remains a controversial entity, with no general consensus about its definition [2]. The phenomenology and duration of seizures as well as their variable electroclinical patterns are the main confounding factors. According to previous data, treatment of NSE is based on the use of phenobarbital (PB) and/or phenytoin (PHT) as first-line agents; however, other drugs have been reported as second-line agents, such as levetiracetam, midazolam, lidocaine, topiramate and vitamins [1,3]. At present, only class III–IV studies with C–D levels of evidence have been published regarding the abovementioned drugs. The use of steroids in the treatment of status epilepticus (SE) in adults or older children has been reported, specifically in refractory status epilepticus [4]. The rationale for its use is mainly based on the modulation of the immune response associated with prolonged epileptic activity leading to short and long-term consequences on neurons and synaptic networks [4]. Here, we report on the successful treatment of NSE with the administration of hydrocortisone in three newborns that failed to respond to conventional agents.

## 2. Materials and Methods

### 2.1. Patient 1

**Figure 1:**
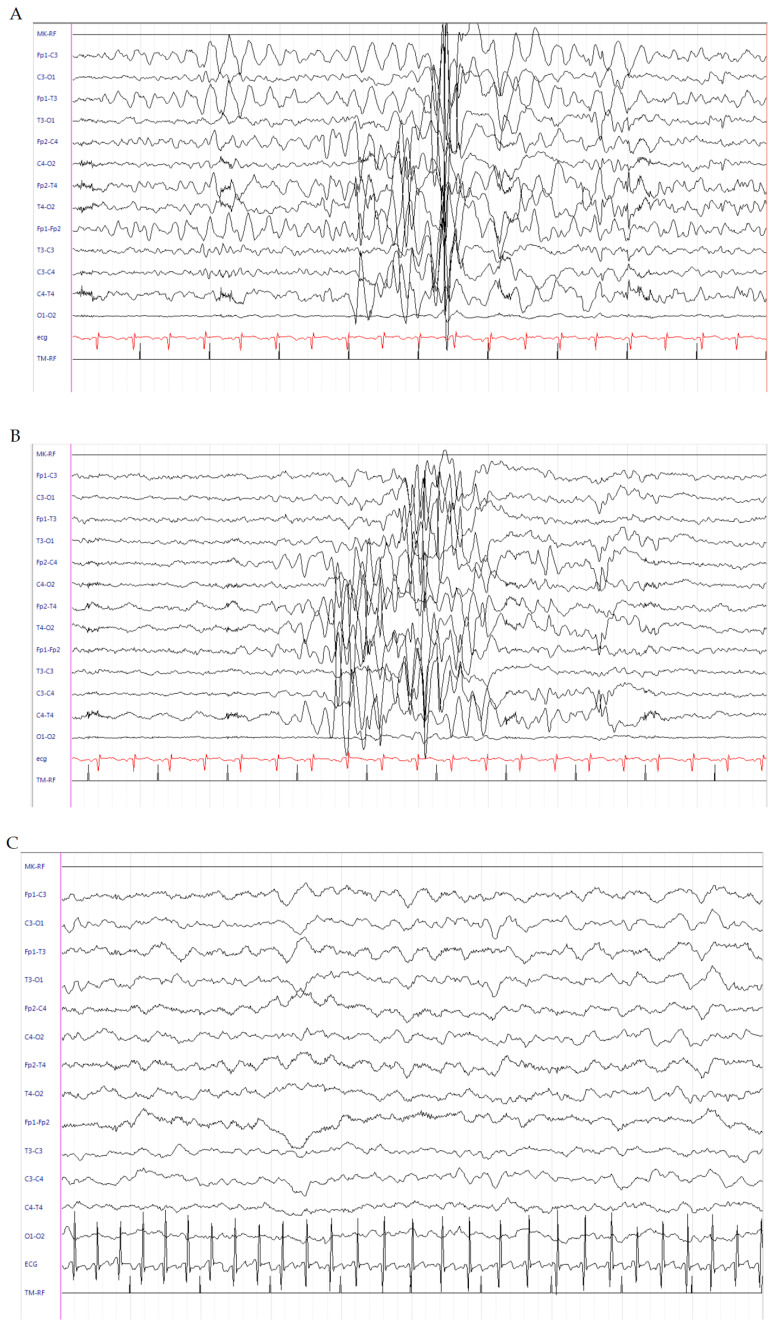
Electroencephalogram (EEG) of patient 1. (**A**) Interictal pattern characterized by onset of high-voltage delta waves in left frontal regions, suddenly spreading to the counter-lateral homologous regions, and (**B**) subsequent generalization, followed by medium–high-voltage delta waves in bilateral centrotemporal regions. (**C**) Background activity mainly characterized by medium–low-voltage theta rhythms.

This is a male patient born at 30^4/7^ weeks of gestational age (GA) from an unremarkable pregnancy, by emergency caesarean section (TC) due to metrorrhagia. Apgar score was 3 at 1 min and 7 at 5 min. Birth weight was 2100 g (>97th percentile). He was admitted to the Neonatal Intensive Care Unit (NICU) with immediate start of noninvasive ventilatory support; however, due to worsening of respiratory conditions, he was intubated and treated with surfactant. Seizures occurred at 3 days old and were characterized by clonic jerks at the lower right limb, often spreading to the right hand and to the counter-lateral limbs, recurring at least 1–2 times/h and lasting for about one minute. Sleeping electroencephalogram (EEG) activity showed an interictal pattern characterized by onset of high-voltage delta waves in left frontal regions, suddenly spreading to the counter-lateral homologous regions (Figure 1A) and subsequent generalization, followed by medium–high-voltage delta waves in bilateral centrotemporal regions (Figure 1B). Interictal neurological examination showed severe diffuse hypotonia and extremely poor general movements. Intravenous (IV) levetiracetam (LEV) (40 mg/kg) as a bolus was administered with only a partial reduction of seizures (occurrence once every 1–2 h). LEV was continued with a maintenance dose of 10 mg/kg/dose three times daily. Pyridoxine as a bolus (100 mg/day) was administered. Due to the seizures persisting, PB (20 mg/kg) as a bolus was administered with transient seizure disappearance; however, they relapsed after 3 h. IV infusion with midazolam was started and uptitrated to 2 μg/kg/min with poor results. Sleeping EEGs were serially performed and showed persistence of electric seizures without relevant clinical manifestations. Cranial ultrasound (CUS) scan failed to detect brain abnormalities. Brain MRI scan was performed, showing eccentric venous thrombosis at the superior right sagittal and left transverse sinuses. Low-molecular-weight heparin (LMWH) therapy was started at a dosage of 150 IU/kg every 12 h [5] with associated folic acid supplementation (100 mcg/day). LEV (60 mg/day) was continued. Pyridoxine (100 mg/day) was suspended due to lack of efficacy. At this time, hydrocortisone (5 mg/kg/day) was started with subsequent clinical and electrical seizure disappearance after 2 days. Interictal EEG abnormalities also disappeared. Brain MRI scan was repeated at days 16 and 27 and showed a regular flow signal in the context of the venous sinuses. LMWH was discontinued. Hydrocortisone was downtitrated 1 mg/kg/day every 3 days up to withdrawal at the corrected age (CA) of 35 + 4 weeks. Continuous IV administration of midazolam was gradually withdrawn. At the time of hospital discharge, the baby was seizure-free and a slight improvement in axial tone was noticed at neurological examination. LEV of 7 mg/kg/dose three times daily was continued. At the follow-up, the patient was evaluated at 38 weeks (CA), where he was seizure-free and thus LEV was downtitrated. A further improvement in axial tone with more variable and complex general movements were evidenced. At the last visit, at 44 weeks CA, the patient was still seizure-free and only showed a mild axial hypotonia with slight hypertonia at the lower limbs. The EEG showed background activity mainly characterized by medium–low-voltage theta rhythms (Figure 1C). A further brain MRI scan was normal. LEV was withdrawn.

### 2.2. Patient 2

**Figure 2:**
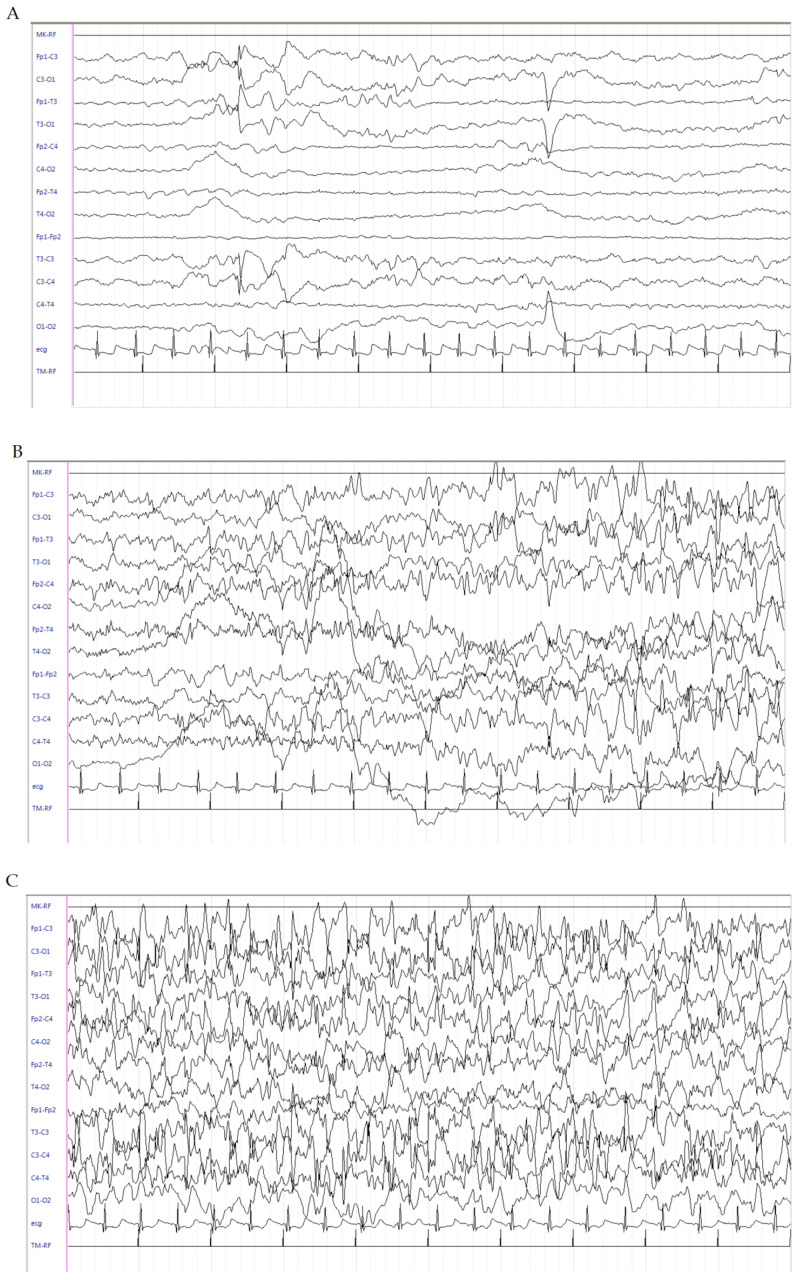

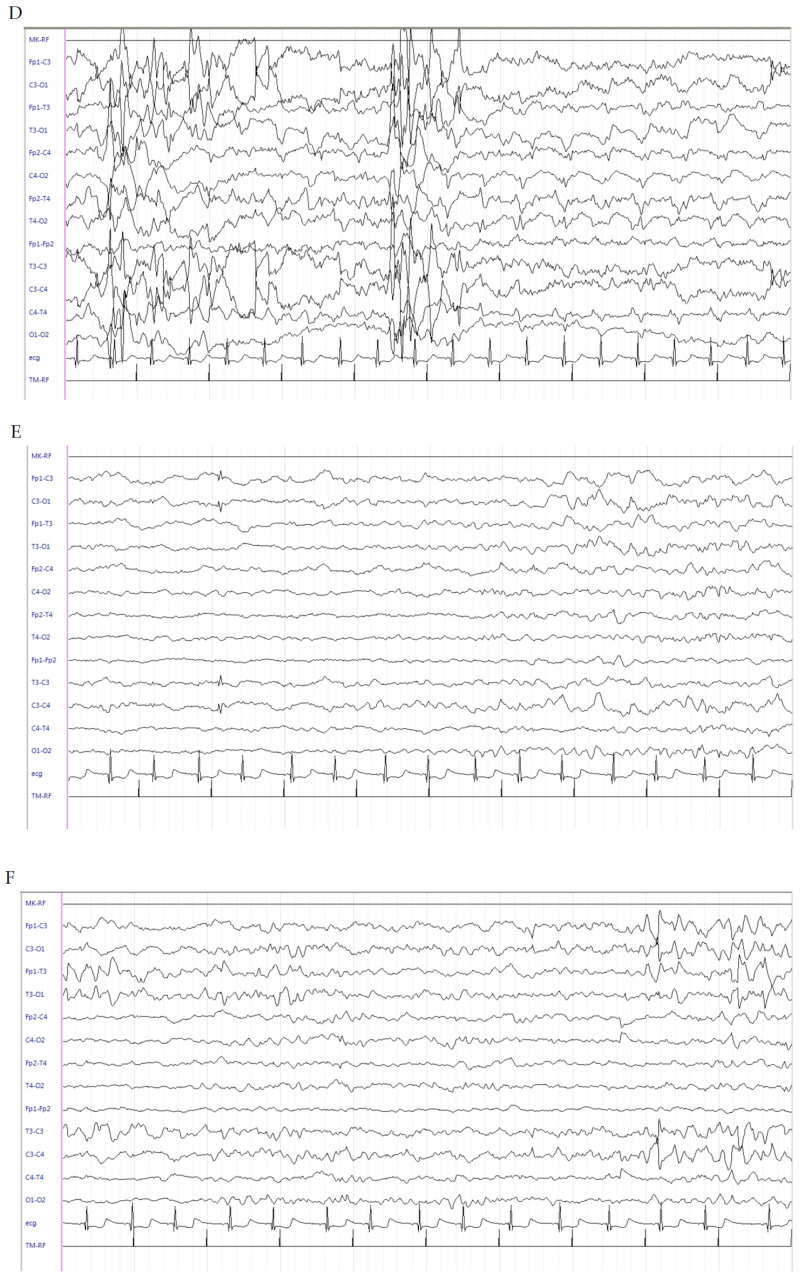

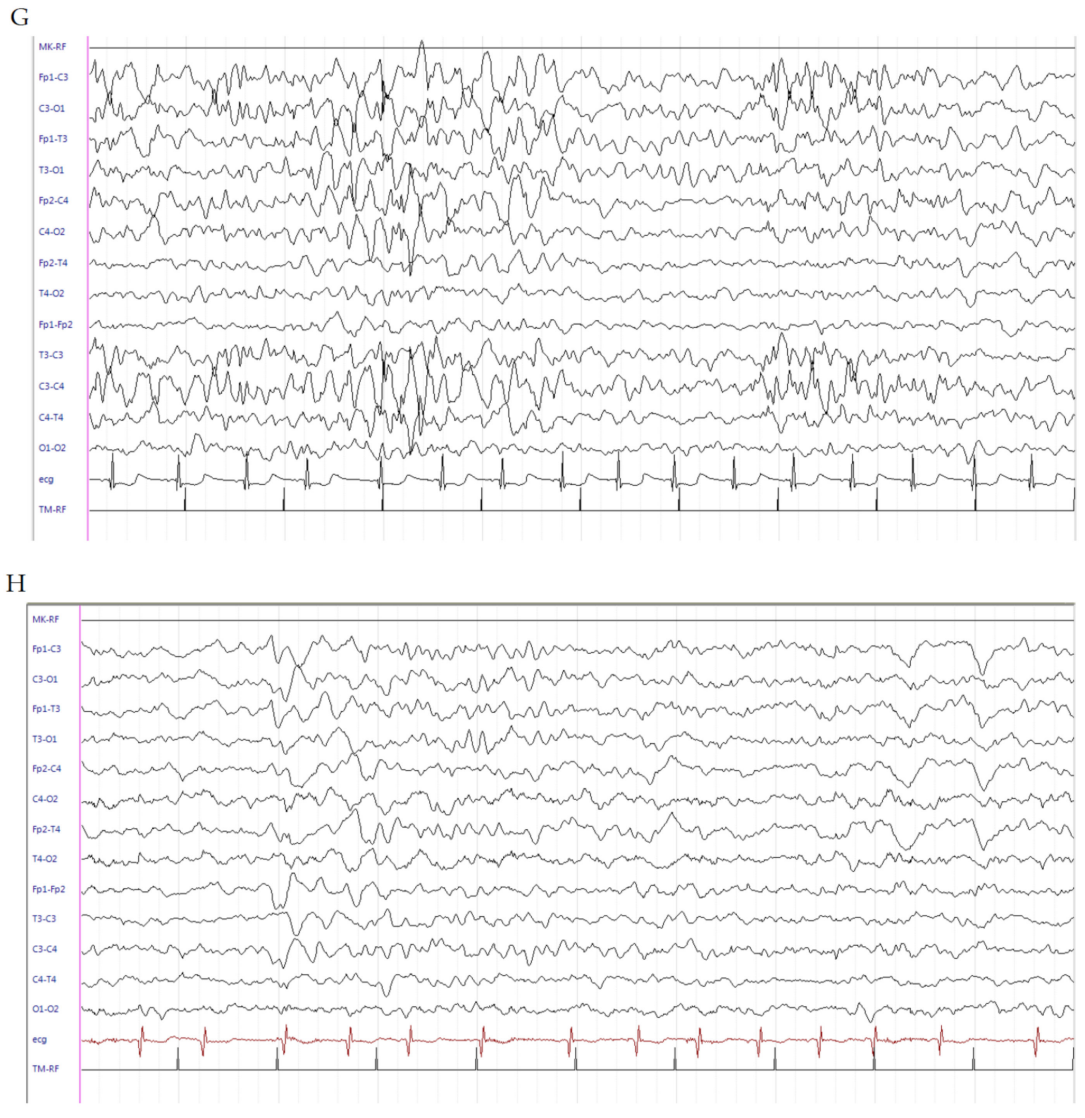
Electroencephalogram (EEG) of patient 2. (**A**–**D**) Ictal EEG shows focal slowing in the left central region, followed by appearance of medium–high-voltage, rapid, spiky activity in the left central region, suddenly generalizing. High-voltage and medium-voltage delta activity mainly in the right central region is evident at the end of the seizure. (**E**,**F**): Interictal EEG recording shows brief medium-voltage spike–slow wave discharges in either the left or right central regions, mostly asynchronous. (**G**): Interictal discharges consisting of prevalent isolated events of brief sharp wave–slow wave complexes turned over to the frontal lobe (left > right). (**H**): Gradual onset of tracé alternant of the newborn.

This is a female patient born at 40^4/7^ weeks of GA from an unremarkable pregnancy by spontaneous vaginal delivery. Her birth weight was 2100 g (<3rd percentile). No perinatal distress was referred, and she was breastfed. On the 3rd day of life, the patient presented with focal seizures mainly characterized by lateralized tonic posturing and flushing, followed by clonic jerks at the same side, later shifting to the contralateral side, associated with perioral cyanosis and sweating lasting almost 30–50 sec, recurring many times a day. Family history was negative for epilepsy and other neuropsychiatric disorders. On the 5th day of life, due to persistence of clinical seizures, lethargy and poor feeding, the child was admitted to the NICU of our hospital. Seizures phenomenology appeared to be heterogeneous with some episodes characterized by generalized hypertonia, numbness and staring; others by focal clonic jerks of the right side; and still others by generalized clonic jerks. The interictal EEG failed to show epileptic discharges. In contrast, the ictal EEG showed focal slowing in the left central region, followed by appearance of medium–high-voltage, rapid, spiky activity in the left central region, suddenly generalizing. High-voltage and medium-voltage delta activity mainly in the right central region was evident at the end of the seizure (Figure 2A–D). Interictal EEG recording shows brief medium-voltage spike–slow wave discharges either in the left or right central regions, mostly asynchronous (Figure 2E,F). The patient underwent LEV 40 mg/kg administered as a bolus, with transient benefit. Oral pyridoxine (100 mg) was administered without benefits. Continuous IV midazolam infusion was kept on and uptitrated to 6 μg/kg/min, with seizure rate reduction but persistence of the interictal discharges consisting of prevalent isolated events of brief sharp wave–slow wave complexes turned over to the frontal lobe (Figure 2G). Extensive routine and metabolic investigations assessed according to previously described methods were unremarkable [6]. Cranial Ultrasound Scan (cUS) and brain MRI scans were normal. For this reason, next-generation sequencing analysis for genetic epilepsies was performed on peripheral leukocyte blood samples of the patient and both her parents, once written informed consent was obtained. On the 9th day of life, a sleeping EEG showed the presence of electroclinical episodes with slight motor and dysautonomic phenomenology. The interictal EEG showed focal discharges apparently shifting from one side to the other, sometimes interspersed with brief tracts of diffuse flattening. LEV was increased to 10 mg/kg/dose three times daily without benefit. PB 20 mg/kg was administered as a bolus with a transient reduction of seizures, but with subsequent relapse and persistence of interictal discharges. On the 10th day of life, therapy with hydrocortisone (5 mg/kg) was started, with seizures disappearing after 24 h. The EEG showed gradual disappearance of the abnormal discharges with gradual onset of physiologic tracé alternant of the newborn (Figure 2H). A gradual withdrawal of midazolam was performed. On the 21st day of life, hydrocortisone was gradually decreased and subsequently withdrawn on the 29th day of life. At this time, neurological examination showed axial hypotonia and poor spontaneous motility, which were markedly improved at the time of the patient’s discharge at 1 month of age. At the 3-month follow-up, the neurological examination showed persistent slight axial hypotonia and mild functional hand asymmetry at the right side. The patient continued LEV (150 mg/day) and she was seizure-free. Next-generation sequencing revealed the presence of a de novo heterozygous mutation in the KCNQ2 gene (c.2555delC (p.Pro852Argfs*78)). At the 6-month follow-up, the patient was seizure-free and the EEG showed high-voltage theta frequencies with sporadic rapid activity in the frontal regions and continued LEV therapy (150 mg/day). Neurological examination was unchanged.

### 2.3. Patient 3

**Figure 3:**
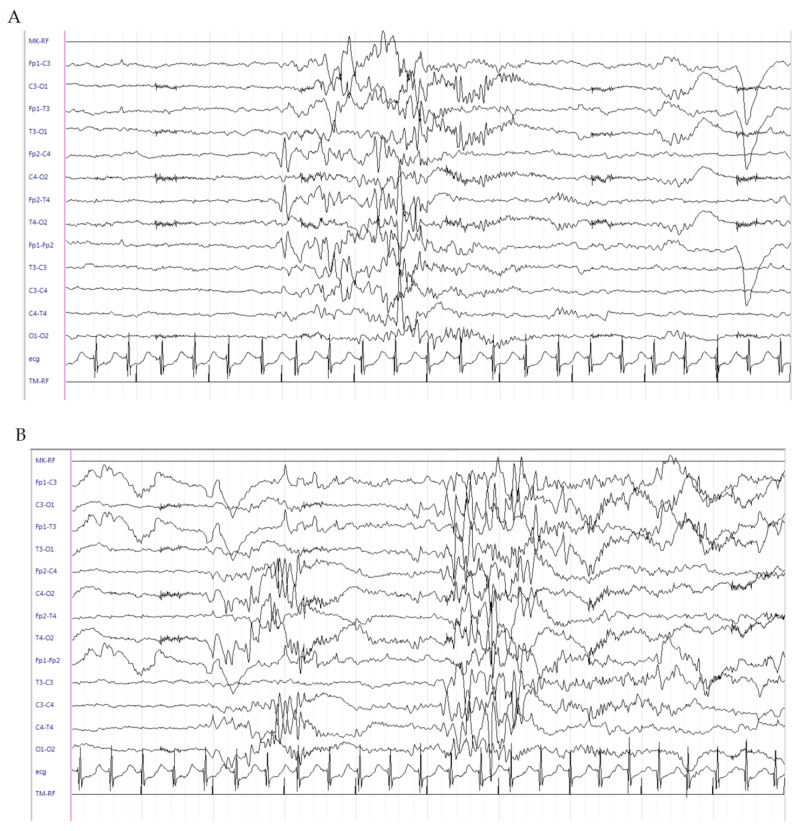

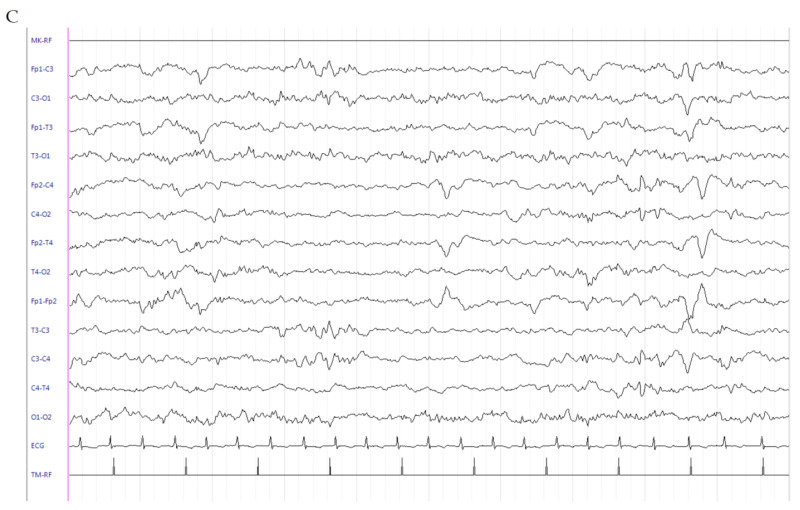
Electroencephalogram (EEG) of patient 3. (**A**) Multifocal medium–high-voltage generalized polyspike discharges. (**B**). Ictal EEG characterized by onset of medium-voltage, rapid, spiky activity in bilateral central regions followed by medium–high-voltage generalized polyspike discharges (**C**). Interictal EEG during quiet sleep recorded at 38 W CA, showing periodic brief sequences of variable activity; low-voltage, alpha-like activity in the central regions; tracts of diffuse background attenuation; and high-voltage sharp waves in the central regions. No epileptiform discharges were evidenced.

This is a male patient born at the 32^4/7^ weeks of GA from pregnancy complicated by threatened abortion since the 21st week of gestation and requiring a cesarean section due to premature rupture of membranes. Apgar score was 7 at 1 min and 9 at 5 min. Birth weight was 1760 g (50th percentile). On the 1st day of life, due to the appearance of left pneumothorax, thoracic drainage was positioned and the baby was admitted to the NICU of our hospital. Noninvasive ventilatory support was maintained with subsequent treatment with surfactant. EV dopamine and adrenaline were administered. However, due to worsening of respiratory conditions, he was intubated. Laboratory tests revealed metabolic acidosis (pH 7.19, PCO_2_ 41.7 mmHg, PO_2_ 49.9 mmHg, EB -12 mEq/L, HCO_3_ 15.4 mEq/L). CUS scan failed to reveal structural abnormalities. Since day 16, daily recurrent seizures (1–2 times/h) appeared with bradycardia, sucking automatisms, clonic jerks and hypertonia to the four limbs, lasting for several seconds. LEV as a bolus was administered (40 mg/kg) and subsequently maintained at 10 mg/kg/dose three times daily. Midazolam as a continuous infusion was gradually uptitrated to 2 mcg/kg/min. Upon neurological examination, the patient was extremely hypotonic with persistent sporadic clonic movements at the four limbs and dystonic posturing elicited by mild tactile stimulation. The EEG showed multifocal epileptic discharges (Figure 3A). Despite the persistence of seizures, due to the severe hypotonia and lethargy of the newborn with frequent ictal bradycardia, PB and PHT treatments were avoided. At this time, hydrocortisone was started (5 mg/kg). A gradual reduction until the disappearance of clinical seizures was observed after 2 days of therapy, and midazolam was gradually suspended. The ictal EEG was characterized by onset of medium-voltage, rapid, spiky activity in bilateral central regions followed by medium–high-voltage generalized polyspike discharges (Figure 3B). No clinically evident seizures have been detected since. Brain MRI scan at the corrected age (CA) of 35 + 4 weeks showed multiple ischemic lesions affecting bilateral subcortical and periventricular white matter with interspersed cavitations. The patient started a gradual decrease of hydrocortisone until withdrawal at 38 weeks (CA). At this age, he was seizure-free and the interictal EEG during quiet sleep recorded at 38 weeks CA showed periodic brief sequences of variable activity, low-voltage, alpha-like activity in the central regions, tracts of diffuse background attenuation and high-voltage, sharp waves in the central regions. No epileptiform discharges were evidenced (Figure 3C). LEV at 60 mg/day was continued. Neurological examination showed a remarkable improvement of the alertness; in contrast, moderate axial hypertonia and poor pattern of general movements were persistently observed.

## 3. Discussion

Here, we have reported on three newborns with NSE that failed conventional first- and second-line AE therapies and were successfully treated with hydrocortisone. The patients were previously treated with PB, LEV and midazolam, showing persistency of clinical and/or electric seizures. The choice of hydrocortisone as a type of steroid in our three newborns was encouraged by its common use in NICU, although in different conditions such as neonatal hypotension [7] and bronchopulmonary dysplasia in preterm infants [8]. Moreover, a nonsignificant influence on neurodevelopmental outcome at 2 years of age has been reported for low doses of hydrocortisone in extremely preterm infants [9]. Neonatal seizures still represent a challenging event, and unfortunately, at present, few drugs are available for their treatment. The role of brain inflammation has been taken into account in the molecular cascade sustaining epileptic status in older children and adults [10,11]; however, it has been less defined in newborns with NSE related to different etiologies [5,12,13,14]. Indeed, the influence of oxidative stress as a risk factor for brain injury in newborns is well known, especially in preterm infants where there is an imbalance deriving from the overproduction of free radicals and the insufficient levels of antioxidant enzymes [15,16]. In particular, inefficient endogenous anti-inflammatory control of brain inflammation has been described both during chronic recurrent seizures and in SE, and in turn, prolonged inflammation has been suggested to sustain epileptic activity in neurons [10]. According to these events, anti-inflammatory treatments either during SE or shortly after SE has elapsed appeared to reduce the severity of the ensuing epilepsy in animal models, with prevention of seizure progression and decreased neuronal loss [17]. The increase of several proinflammatory cytokines, such as IL-1β, TNF-α and IL-6, may contribute to the decrease of seizure threshold in animal models, either by activating their neuronal receptors, thereby rapidly affecting neuronal excitability, or by slower onset effects related to the transcriptional activation of genes involved in synaptic and molecular plasticity [4]. These effects have been mostly studied in developing rats or mice exposed to SE at post-natal days 7–14, representative of the perinatal/early infancy period in humans. Inflammation evoked by seizures or SE in the developing brain is unique compared with the adult brain, since inflammatory mediators play a role in normal development by regulating neurogenesis and synapse formation [18,19]. As a rule, the developmental timelines may affect how the brain will respond to seizures [20]; thus, an excess of seizure-induced inflammatory signaling during brain maturation has the potential to interfere with neurodevelopmental processes [21]. The mechanisms underlying the long-lasting increase in brain excitability due to prolonged seizures are not well understood yet, although the involvement of glutamate and GABA receptor levels and their subunit composition, as well as the Na+ -K+ -2Cl- cotransporter in the forebrain, have been previously taken into account [9]. Similarly to our study, Verhelst et al., described a series of pediatric patients treated with hydrocortisone and other steroids for intractable epilepsy; in particular, 10 of out 36 patients were treated with hydrocortisone, and five of them became seizure-free, while one out of 10 showed a 25% reduction in seizures. However, none of these patients were treated with hydrocortisone in the neonatal period, as in our study [22]. Our patients presented with clinical and/or electrical seizure remission after hydrocortisone therapy, independently from the underlying etiology. As it could be expected, patient 3, with severe diffuse brain hypoxic-ischemic damage, showed a slower response, with disappearance of clinical seizures after 2 days despite persistence of multifocal discharges in the EEG up to 2 weeks after starting hydrocortisone. Furthermore, treatment of patient 2, with a normal brain MRI scan, resulted in the identification of a pathogenic mutation in the KCNQ2 gene, commonly associated to benign familial neonatal convulsions (BFNCs). This is a rare autosomal dominant disorder, mainly familial, characterized by recurrent and prolonged seizures with onset within the first days of life. The interictal EEG is commonly unremarkable. Seizures usually cease with carbamazepine treatment within days to some weeks, and the patients show normal psychomotor development [23]. In contrast, a type of KCNQ2-related early-onset epileptic encephalopathy has been reported, with neurodevelopmental impairment and subsequent seizures [23]. Although BFNCs were taken into account in the diagnostic workup of patient 2 and genetic analysis was immediately performed, some features of the patients were quite discordant with the typical BFNCs frame. The baby showed nonfamilial seizures, seizure phenomenology was heterogeneous, neurological examination was definitely abnormal and the interictal EEG pattern showed periodic multifocal discharges with frequent side shifting resembling those of an early-onset epileptic encephalopathy. At least, according to all these features, we felt encouraged to start hydrocortisone therapy, and we are still observing the baby throughout the follow-up to further define her long-term electroclinical evolution. In conclusion, although this is a small case series, reconsideration of the treatment of NSE is worthwhile to be taken into account in multicenter studies, also including adjunctive nonconventional therapies such as steroidal anti-inflammatory drugs. Moreover, further investigations about the reactivity of the immature brain to prolonged epileptic activity with the identifications of specific molecular pathways may help to reach ideally targeted therapies.
